# Viremic HIV Infected Individuals with High CD4 T Cells and Functional Envelope Proteins Show Anti-gp41 Antibodies with Unique Specificity and Function

**DOI:** 10.1371/journal.pone.0030330

**Published:** 2012-02-01

**Authors:** Marta Curriu, Hughes Fausther-Bovendo, María Pernas, Marta Massanella, Jorge Carrillo, Cecilia Cabrera, Cecilio López-Galíndez, Bonaventura Clotet, Patrice Debré, Vincent Vieillard, Julià Blanco

**Affiliations:** 1 IrsiCaixa-HIVACAT, Institut de Recerca en Ciències de la Salut Germans Trias i Pujol (IGTP), Hospital Germans Trias, Universitat Autònoma de Barcelona, Badalona, Barcelona, Catalonia, Spain; 2 INSERM UMR-S 945, Laboratoire Immunité et Infection, Hôpital Pitié-Salpêtrière, UPMC, Université Paris-6, Paris, France; 3 Centro Nacional de Microbiología (CNM), Instituto de Salud Carlos III, Majadahonda, Madrid, Spain; 4 Lluita contra la SIDA Foundation, Institut de Recerca en Ciències de la Salut Germans Trias i Pujol, Hospital Universitari Germans Trias i Pujol, Universitat Autònoma de Barcelona, Badalona, Barcelona, Spain; George Mason University, United States of America

## Abstract

**Background:**

CD4 T-cell decay is variable among HIV-infected individuals. In exceptional cases, CD4 T-cell counts remain stable despite high plasma viremia. HIV envelope glycoprotein (Env) properties, namely tropism, fusion or the ability to induce the NK ligand NKp44L, or host factors that modulate Env cytopathic mechanisms may be modified in such situation.

**Methods:**

We identified untreated HIV-infected individuals showing non-cytopathic replication (VL>10,000 copies/mL and CD4 T-cell decay<50 cells/µL/year, Viremic Non Progressors, VNP) or rapid progression (CD4 T-cells<350 cells/µL within three years post-infection, RP). We isolated full-length Env clones and analyzed their functions (tropism, fusion activity and capacity to induce NKp44L expression on CD4 cells). Anti-Env humoral responses were also analyzed.

**Results:**

Env clones isolated from VNP or RP individuals showed no major phenotypic differences. The percentage of functional clones was similar in both groups. All clones tested were CCR5-tropic and showed comparable expression and fusogenic activity. Moreover, no differences were observed in their capacity to induce NKp44L expression on CD4 T cells from healthy donors through the 3S epitope of gp41. In contrast, anti- Env antibodies showed clear functional differences: plasma from VNPs had significantly higher capacity than RPs to block NKp44L induction by autologous viruses. Consistently, CD4 T-cells isolated from VNPs showed undetectable NKp44L expression and specific antibodies against a variable region flanking the highly conserved 3S epitope were identified in plasma samples from these patients. Conversely, despite continuous antigen stimulation, VNPs were unable to mount a broad neutralizing response against HIV.

**Conclusions:**

Env functions (fusion and induction of NKp44L) were similar in viremic patients with slow or rapid progression to AIDS. However, differences in humoral responses against gp41 epitopes nearby 3S sequence may contribute to the lack of CD4 T cell decay in VNPs by blocking the induction of NKp44L by gp41.

## Introduction

HIV infection is characterized by an important decrease on CD4 T cell count, resulting in weakened immune responses that lead to AIDS-defining events. Progression to AIDS among HIV-infected individuals is highly heterogeneous due to host and viral factors [1], [2], ranging from <3 years in rapid-progressors (RP) to >10 years in long term nonprogressors (LTNP). Usually, LTNPs show undetectable or controlled (<2000 copies/ml) HIV replication; however, a reduced number of LTNP show uncontrolled viral load (VL>2,000 copies/ml) with asymptomatic HIV infection over almost 10 years after seroconversion [1]. Furthermore, a really limited group of HIV-infected individuals show a particular discordant profile with high viral load (VL>10,000 copies/ml) in the absence of quantitative immune defects (Viremic Non-Progresors, VNP). This fact is paradoxical, as HIV-infected CD4 T lymphocytes have a shortened lifespan due to direct cytopathic effects of HIV [3] or lysis by immune cells [4]. Moreover, the number of dying cells in infected individuals greatly exceeds the number of HIV-infected cells [4] due to detrimental effects of immune activation [4], HIV proteins [5], [6] or abortive infection [7] on the bystander uninfected CD4 T cell population. Among viral determinants, the envelope glycoprotein (gp120/gp41, Env), which defines HIV tropism for CCR5 or CXCR4, can influence CD4 T cell decline in vitro [8] and in vivo [9]. Furthermore, Env is a major determinant of viral pathogenicity, which is related to the fusogenic activity of gp41 [10], [11] and affects both infected [12] and bystander CD4 T cells [13]–[15].

This plethora of cytopathic mechanisms of HIV seem to fail in particular SIV-infected primates (sooty mangabeys) and in a small subset of VNP patients, showing constant level of CD4 T cells despite high-level viral replication [16], [17]. Several attempts to unravel this paradox have pointed to strong differences in the level of immune activation [17], [18], CCR5 expression in GALT [16] or the expression of NK activating ligands [19] among individuals showing pathogenic versus non pathogenic HIV replication as non-excluding reasons for the different outcome of infection.

It has been proposed that CD4 T cell depletion is, partly, a consequence of the expression of the NK ligand NKp44L on CD4 T cells, which render these cells sensitive to NK lysis [20]. Interestingly, NKp44L is induced by the gp41 HIV envelope glycoprotein. Indeed, a highly conserved motif in gp41, called 3S, plays a critical role in the translocation of NKp44L to the surface of CD4 T cells [20] by engaging the receptor for the globular domain of C1q (gC1qR) on these cells [21]. The NKp44L cell surface expression correlates with the extent of CD4 T cell depletion and is inhibited by humoral responses against the 3S epitope in both HIV-infected individuals and SHIVinfected macaques [22]–[24]. Moreover, the disappearance of anti-3S antibodies over progression to AIDS is concomitant with CD4 T cell depletion and with an increase in the expression of NKp44L on the surface of these cells [19],[22].

We have identified a small group of VNPs who display high constant CD4 T cell counts despite continuous active viral replication. Given the multifaceted role of HIV Env in cytopathic events, we analyzed full-length envelope clones isolated from these patients. The role of viral tropism, fusion activity, expression of NKp44L on CD4 T cells and the presence of protective anti-gp41 antibodies have been evaluated and compared with RPs.

## Materials and Methods

### Individuals

Four viremic non-progressors (VNP) without antiretroviral therapy during at least two years (and naive for fusion inhibitors) were identified in the Hospital Germans Trias i Pujol (Badalona, Spain) fulfilling selection criteria for non-cytopathic HIV high replication: documented VL>10.000 copies RNA/mL and levels of CD4 T cells >400 cells/µl with a loss of CD4 T cells <50 cells/µl/year. For comparative purposes, a matched group of five Rapid Progressors (RP) was selected from Centro Sanitario Sandoval (Comunidad Autónoma de Madrid). Rapid progression was defined by CD4 T cell levels <350 cells/µl within 3 years after seroconversion, documented by a HIV negative test within one year before the first positive test. All procedures followed the Helsinki Declaration in 1975, as revised in 1983, and were approved by the Ethics committee of the Hospital Germans Trias I Pujol. All individuals provided their written informed consent. Plasma and peripheral blood mononuclear cells (PBMC) were obtained from selected patients by standard protocols and cryopreserved until use.

### Cells, Reagents and plasmids

Samples from HIV negative healthy individuals were obtained at the local blood banks. Buffy-coats were processed to obtain PBMC, which were immediately used to purify CD4+ T cells (>95%) by immunomagnetic positive selection (Miltenyi-Biotec). 293T cells (ATCC) and TZM-bl cells (NIH AIDS Research and Reference Reagent Program) were maintained in DMEM supplemented with 10% heat inactivated Fetal Bovine Serum (FBS) with selection antibiotics when required. All media were from Invitrogen.

Anti-CD4 monoclonal Antibody (mAb) Leu3a was from BD Biosciences; anti-gp120 IgGb12 and 2G12 mAbs, and anti-g41 4E10 and 2F5 mAbs were from Polymun; 3S peptide, anti-3S and anti-NKp44L antibodies have been previously described [19], [22]. CCR5 and CXCR4 antagonists TAK-779 and JM-2987 respectively, were from NIH AIDS Research and Reference Reagent Program.

Expression plasmids coding for SVBP13, SVBP16 [25] and BaL.1 [26] envelopes, the Env-defective pSG3 plasmid [26] and the tat expression plasmid pTat [27] were obtained through the NIH AIDS Research and Reference Reagent Program. Env from the NL4-3 isolate was amplified from pHenv plasmid [28] and cloned as described below.

### HIV envelope amplification and cloning

Virion-associated RNA was purified from plasma samples (QIAmp viral RNA, QIAGEN). Full-length *env*/*rev* genes were amplified as described [25]. PCR products from position 5954 to 8904 (HXB2 numbering) were purified using the SNAP kit (Invitrogen) and cloned into the pcDNA3.1D/V5-His Topo vector (Invitrogen). 10–15 positive transformant bacterial colonies were isolated from each patient.

### Genotypic characterization of HIV Env

The V3 loop of gp120 and the gp41 ectodomain were sequenced using specific primers and BigDye Terminator v3.1 kit (Applied Biosystems) with an automated DNA Sequencer (3100 Analyzer; Applied Biosystems). Sequences were edited using the Sequencher program 4.26 (Gene Codes Corp.). To assess the tropism of the Env clones, the V3 loop sequences were analyzed by both PSSM [29] and geno2pheno software [30]. All sequence data has been deposited in GenBank with accession number JN673277 to JN673310 (3S epitope sequences) and JN673311 to JN673335 (V3 loop sequences).

### Analysis of HIV Env-mediated fusion

293T cells were co-transfected with pTat and Env clones using CalPhos (Clontech). As negative control, 293T cells were transfected only with pTat. 293 T cells were chosen as effector cells since they provide sensitive measures of fusion even when using low fusogenic envelopes (Cunyat F, Curriu M et al, Submitted). 24 hours post-transfection, cells were collected, and tested for Env surface expression and fusion activity. NL4-3 and BaL Env expression plasmids were used as positive control for Env staining and as reference value for fusion activity (BaL = 100%). To test Env expression, 2×105 Env-Tat transfected 293T cells were incubated with 2G12 and IgGb12 mAbs at 4 µg/ml (each) for 40 minutes at 37°C. After washing the cells, the PE-labeled goat anti-human IgG (Jackson ImmunoResearch Laboratories) was added and incubated at room temperature for 15 minutes. Cells were washed, fixed in formaldehyde 1%, acquired in a FACS LSRII flow cytometer and analyzed by the Flow-Jo software (Tree Star Inc.) The percentage of Env-expressing cells and the Mean Fluorescence Intensity (MFI) of these cells were considered as individual parameters or used to calculate Relative Fluorescence Intensity (RFI = % of Env+cells×MFI of Env+cells) as described [31].

To test fusion activity, 1×104 Env/Tat-transfected or control Tat-transfected 293T cells were mixed (ratio 1∶1) in 96-well plates with CD4+CXCR4+CCR5+TZM-bl reporter cells for 6 hours. Luciferase activity was measured (Fluoroskan Accent, Labsystems) using Brite-Lite (PerkinElmer) and normalized to BaL-mediated fusion. To obtain a phenotypic measure of tropism and corroborate in silico results, fusion was also assayed in the presence of TAK779 and JM-2987 (1 µg/ml). A Fusogenicity Index was calculated for each Env as the ratio between the fusion activity value obtained in the absence of drugs and the Relative Fluorescence Intensity.

### Pseudovirus production and NKp44L induction assay

Pseudoviruses with different Env were obtained by co-transfection of 293T cells with Env expression vectors and pSG3 plasmid (Calphos). Supernatants were collected 36 hours post-transfection, titrated in TZM-bl cells and stored at −80°C until use.

Purified CD4 T cells from healthy donors were activated in RPMI medium with 10% FBS, 5 µg/mL PHA (Sigma-Aldrich) and 10 U/mL IL-2 (Roche) during 72 hours and then maintained with 10 U/mL IL-2. Activated cells were incubated for 5 hours at 37°C with 3S peptide (5 µg/ml) or different pseudoviruses (1,000 TCID50/ml) that have been preincubated in the presence or the absence of anti-3S antibodies or autologous plasma (from 1/50 to 1/5,000 dilutions). Cells were then stained with anti-NKp44L mAb, washed, stained with PE-Rat anti-mouse IgM (BD Biosciences) and analyzed by flow cytometry (LSRII, BD Biosciences). For the analysis of the expression of NKp44L in PBMC from HIV infected individuals, unstimulated thawed cells were stained with anti CD3 and anti CD4 antibodies and the cell surface expression of NKp44L was stained as indicated above. Alternatively, cells from VNP individuals were treated with a synthetic 15-mer peptide containing the 3S sequence, (consensus peptide V, see below), prior to staining. In both cases, gated CD3+CD4+ cells were analyzed.

In some experiments, IgG were purified from plasma samples using the Ab-Spin Trap kit (GE Healthcare, Madrid, Spain) following manufacturer's instructions. IgG-depleted plasma and dialyzed IgG preparations were stored at −80°C until use. Protein content in IgG purified fractions was assessed by the Pierce BCA protein assay kit (Thermo Scientific, Rockford, IL).

### Analysis of humoral reponses

Quantification of antibodies recognizing the 3S epiotpe or its flanking regions was performed by ELISA as described [22] and expressed in arbitrary units (AU). The peptides used in this study (Covalab, France) were derived from patient sequences: NH2-PWNSSWSNKSYEQIW–COOH (VNP-8), NH2-PWNTSWSNKTLNDIW – COOH (VNP-9), NH2- PWNASWSNKSLNDIW-COOH (VNP-11), NH2- PWNTSWSNKSYHEIW–COOH (VNP-16) or from consensus sequences NH2- PWNASWSNKSLDDIW–COOH (consensus peptide V). For HIV neutralization assays, 200 TCID50 of pseudoviruses bearing NL4-3, BaL, SVBP13 and SVBP16 envelopes (obtained as described above) were pre-incubated for 1 h at 37°C, in 96 well plates with control reagents Leu3a (0.25 µg/ml), IgGb12, 4E10 and 2F5 (10 µg/mL) or plasma dilutions (range 1/60-1/1620) and added to duplicate wells containing 10,000 TZM-bl cells and 37.5 µg/ml DEAE-dextran (Sigma). Cultures were analyzed after two days by luminometry as described above.

### Statistical analysis

Variables were compared using non-parametric tests. Non-linear fit was used to calculate IC50 values of HIV neutralization and blockade of NKp44L induction. Normalized values fitted to an one-site inhibition curve with fixed Hill slope [32]. For IC50 calculations in neutralization assays, plasma samples without detectable inhibitory capacity were considered to have an IC50 of reciprocal dilution of 60. All statistical analyses and non-linear fitting were performed using the GraphPad Prism v5.0 software.

## Results

### Patient description

**Figure 1:**
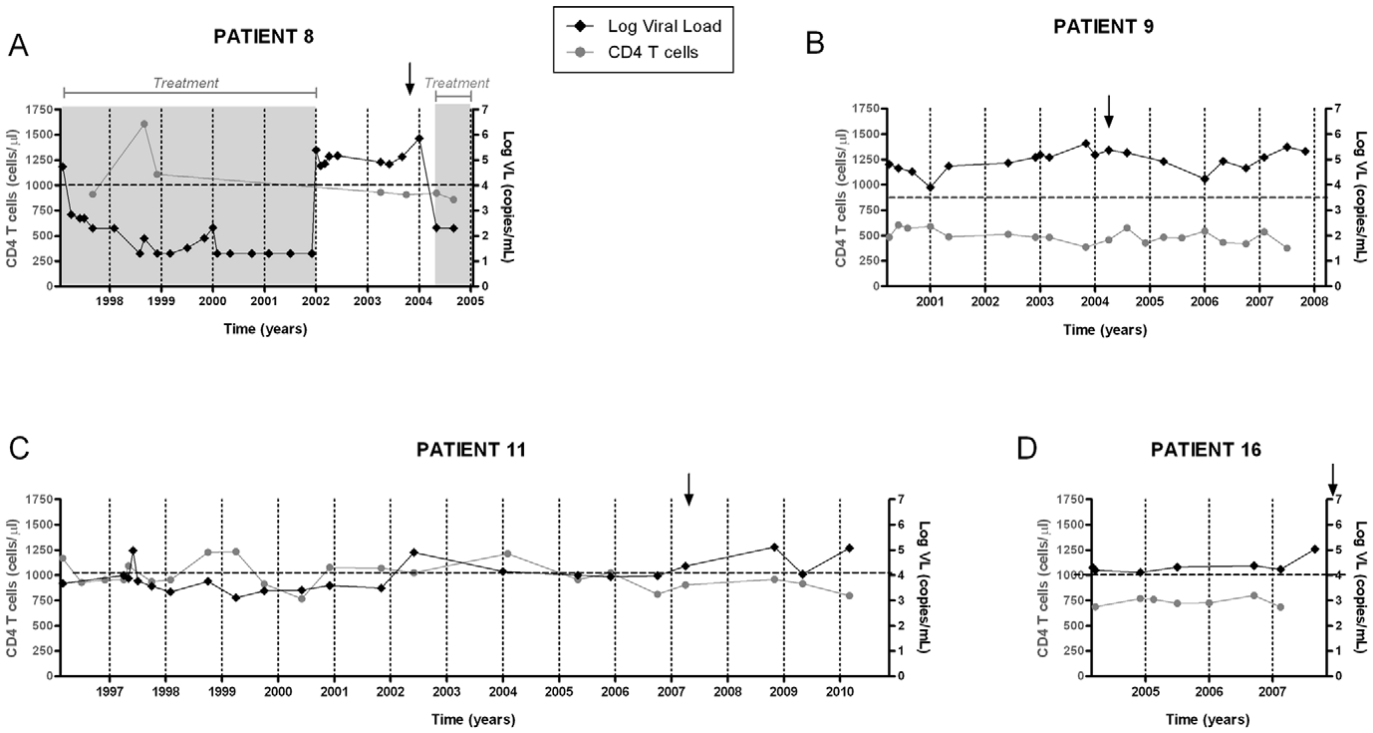
Evolution of VL and CD4 T cell counts in selected viremic non-progressor patients. The time course of absolute CD4 T cell counts (grey circles) and plasma VL (black diamonds) are plotted for the different periods off treatment corresponding to patients 8,9,11 and 16. For patient 8 the pre and post treatment periods are also depicted (grey areas). Arrows indicate plasma samples used for Env cloning.

Four VNP individuals were identified. Figure 1 shows their VL and CD4 T cell count longitudinal evolution. Mean CD4 T cell count for patients VNP-8, 9, 11 and 16 was 945, 486, 995 and 735 cells/µl, respectively (Figure 1). Mean VL for patients VNP-8, 9, 11 and 16 was 168193, 126383, 25144 and 31523 copies RNA/ml (Figure 1). Individuals VNP-8, 9 and 11 showed low CD4 T cells loss, 48, 14 and 6 cells/µL per year, respectively; while VNP-16 showed an increasing trend of 4 cells/µL per year (Figure 1). Individual VNP-8 received antiretroviral therapy from 1997 to 2002, and after 2004. Plasma samples selected for this study belong to year 2003, when the patient was off therapy. Patient VNP-11 showed VL>10,000 copies/ml the last five years of follow up, samples analyzed correspond to this period (Figure 1).

In contrast, individuals identified as RPs showed CD4 T cell counts below 350 (median 262) cells/uL with VL comparable to VNPS (median 77,000 copies/mL). All samples from this group were collected from HAART-naïve patients within 3 years after seroconversion (median 0.7 years).

### Env tropism, fusion capacity and expression

**Figure 2:**
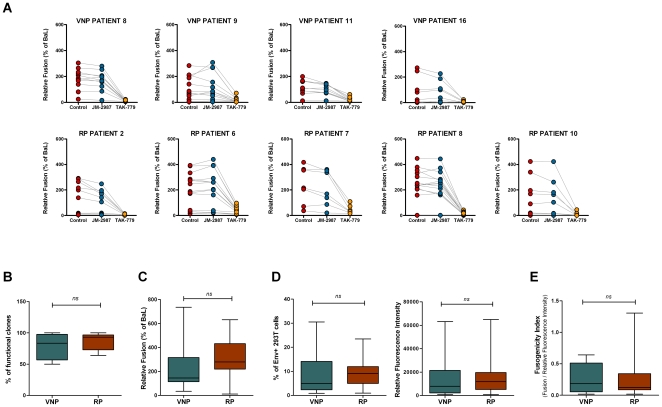
Analysis of Env tropism, fusion and expression. (A) The levels of fusion of functional Env clones isolated from viremic non-progresors (VNP) patients 8, 9, 11 and 16 and rapid progressors (RP) was assayed in a coculture of Env-Tat expressing 293T cells and TZM-bl cells (red symbols). The CXCR4 antagonists JM-2987 or CCR5 antagonists TAK-779 (blue and yellow symbols, respectively) were added as controls for functional tropism. Fusion activity was calculated relative to the Env BaL clone (100%) that was tested in each experiment. Negative controls were obtained by using 293T cells transfected only with a pTat plasmid and were subtracted to all measures. Values for each clone are the mean of three different experiments. (B) The frequency of functional clones was evaluated in both VNPs (green bar, total number of tested clones 44) and rapid-progressor (red bar, total number of tested clones 56) groups. (C–E) Comparative analysis of representative Env clones (n = 32) isolated from VNP (n = 15) and RP (n = 17) patients. (C) Levels of fusion capacity of selected Env clones isolated from VNP and RP patients (green and red bar, respectively) as described for controls on panel A. Fusion activity was calculated relative to the Env BaL clone (100%). (D) Left panel shows the percentage of Env+ cells and right panel shows the Relative Fluorescence Intensity, which is a measure of total envelope expression that accounts for both the percentage of Env expressing cells and the fluorescence intensity of positive cells (see Methods). (E) A fusogenic index was calculated for each selected Env clone as the ratio of Fusion activity and Relative Fluorescence Intensity. For panels B–E, boxes represent median and min-max values.

Full-length envelopes were amplified from plasma samples and cloned into expression vectors. Since Env tropism is a major determinant of cytopathicity [9], V3 loop sequences of the Env clones were analyzed by both PSSM [29] and geno2pheno software [30]. All clones isolated showed a V3 loop sequence predicted as R5-tropic in silico (Table S1). Next, Env clones were screened for fusion activity after cotransfection with pTat into 293T cells and cocultured with TZM-bl reporter cells (Figure 2A). In these experiments we used the R5 BaL envelope clone as positive fusion control (100%) and pTat as negative control (0%). Most of Env clones showed detectable fusogenic activity (>10% of BaL values, Figure 2A). Remarkably, no differences were observed between VNPs and RPs when the percentage of functional clones was analyzed (values ranging from 50% for patient VNP-16 to 100% for patient VNP-8; Figure 2B) Furthermore, the use of co-receptor inhibitors in the fusion assay provided a phenotypic measure of tropism. The lack of effect of JM-2987 and the sensitivity to TAK-779 of all clones isolated from VNPs or RPs confirmed the general use of CCR5 (Figure 2A). Median fusion values for all patients were comparable or higher than the fusion elicited by the BaL Env clone (ranged from 99, 23–273 (median, min-max) % of BaL Fusion for individual VNP-16 to 248, 18–392% for individual RP- 6). Nevertheless, comparison of median fusion values between RPs and VNPs showed a non significant trend towards a lower fusion in the latter group (data not shown). Therefore, to further analyze this potential difference, we selected 4 representative clones from each patient and we characterized their expression and fusogenic capacity. Figure 2C shows that selected Env clones from RPs and VNPs maintained a non significant trend in fusion values (p>0.05) and showed similar levels of expression as measured by the % of Env+ cells or the Relative Fluorescence Intensity, which takes also into account the level of Env expression in positive cells (Figure 2D). Furthermore, the definition of a Fusogenicity Index that normalizes Fusion values to Env expression showed comparable values in both groups of individuals (Figure 2E). As a whole, these data suggest that Env tropism and fusion capacity may hardly explain the very different behavior of CD4 T cells displayed by VNPs and RPs.

### Induction of NKp44L by the gp41 3S epitope

**Figure 3:**
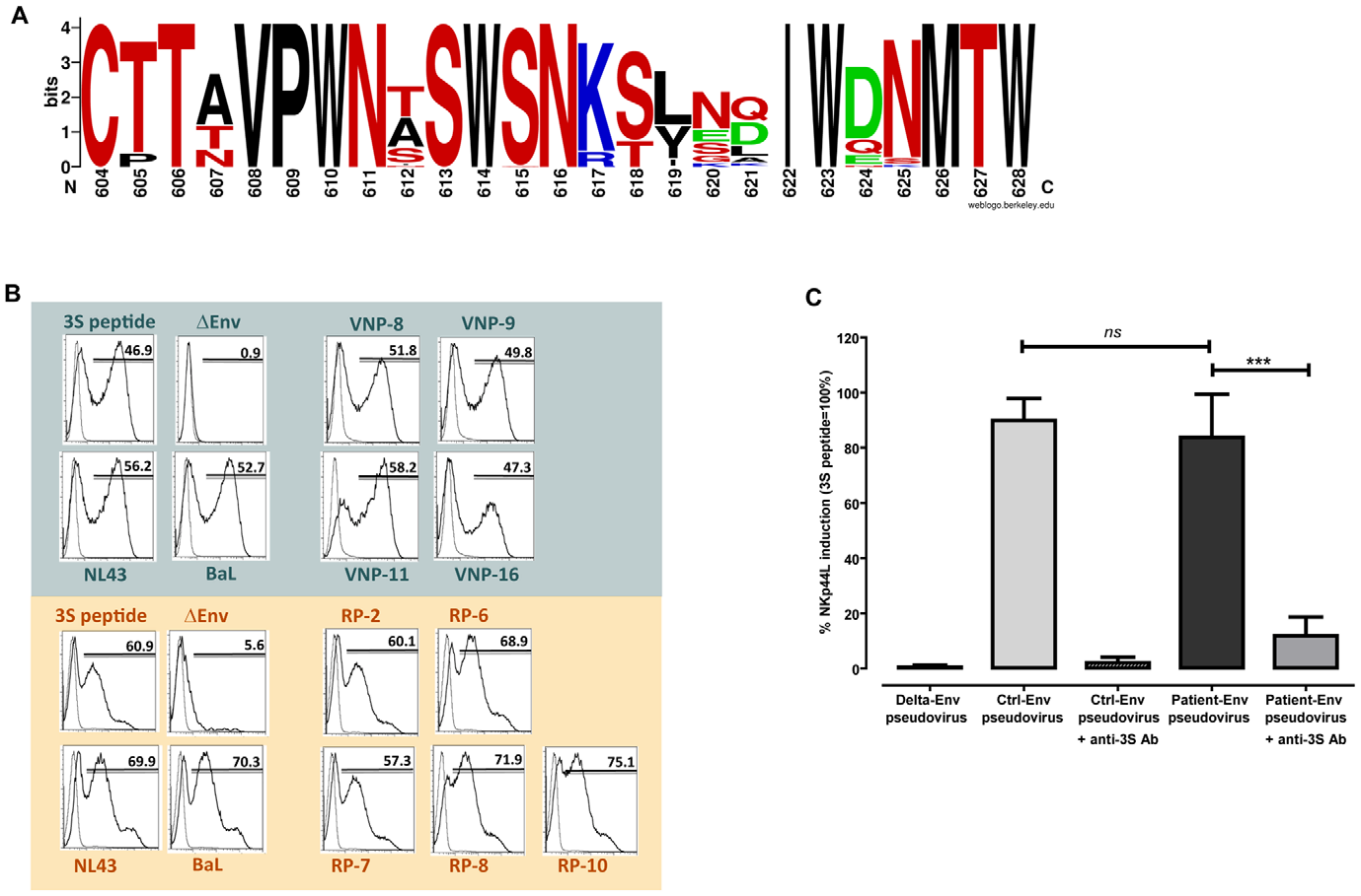
Analysis of the NKp44L induction by cloned envelopes. (A) Graphic representation of sequence variability in gp41 regions surrounding the 3S epitope. The picture has been generated using the weblogo software (http://weblogo.berkeley.edu/), representing polar amino acids in red, basic in blue, acidic in green and hydrophobic in black. (B) NKp44L expression in CD4 T cells from representative donors after incubation with Env-defective pseudoviruses (pSG3, negative control), with the 3S consensus peptide V, or with pseudoviruses bearing the following HIV Env: NL4-3, BaL (positive controls). The effect of selected Env clones from VNPs (upper panels) or RPs (lower panels is shown) Empty peaks correspond to control staining of untreated CD4 T cells. (C) The effect of a polyclonal anti 3S antibody on NKp44L expression induced by several Env clones is shown. Values represent the relative expression of NKp44L in the surface of CD4 T cells normalized to the effect of the 3S synthetic peptide (100%). Data show the effect of NL4-3 and BaL envelopes (Ctrl-Env) and a total of 8 patient-derived Env clones.

To investigate alternative Env functions that determine CD4 T cell loss, we analyzed genotipically and phenotipically the 3S motif of gp41, residues 613–618 [20]. First, sequence analysis revealed the absence of mutations that could impair NKp44L induction in CD4 T cells (Table 1). Sequence was patient dependent, all clones from patient VNP-8 and 16, were wild-type (SWSNKS), while patients VNP-9 and 11 displayed conservative S/T and K/R changes, respectively, in all or some Env clones. In RPs, patients 2 also showed conservative substitutions (K/R or S/T), although a minor clone showed non-conservative point mutations. Most of these relatively high frequent polymorphisms (13-10% in subtype B isolates) have been described to maintain the function of the wt sequence SWSNKS [20]. A wider analysis showed that the 3S epitope is surrounded by regions with higher inter- and intra-patient variability (Table 1), being the highest number of changes located in residues 607 and 612 and residues 619–621, all of them juxtaposed with the 3S epitope (Figure 3A).

**Table 1:** Genotypic characterization of 3S region.

		Frequency
*HXB-2:*	600 610 620 630................A..... ...... .EQ..NHT.....D...N....	
Cons. B	QLLGIWGCSGKLICTTTVPWNA SWSNKS LDEIWDNMTWMEWEREIDNYTS	
**VNP-8**	**..........R.....A....S ...... YEQ..................R**	55 % (6/11)
	**...................... ...... YKQ..................R**	18 % (2/11)
	**.....................S ...... QKQ..E................**	9 % (1/11)
	<1emph type="bold">..........R.....A....S ...... YEQ..E...............R	9 % (1/11)
	**..........R.....A....S ...... YEQ..........K.......R**	9 % (1/11)
		
**VNP-9**	**................A..... .....T .ND........Q..K......N**	50 % (5/10)
	**................A..... .....T .ND..................N**	40 % (4/10)
	**................A..... .....T .ND........Q.........N**	10 % (1/10)
		
**VNP-11**	**.R..........V...N..... ...... .ND...S....Q..K......G**	22 % (2/9)
	**.R..........V...N..... ...... .ND...K....Q..K......G**	22 % (2/9)
	**.R..............N..... ...... .ND...S....Q..K......G**	22 % (2/9)
	**.R..........V...N..... ....R. .ND...N....Q..K......G**	22 % (2/9)
	**.R..........V...N..... ...... .NA...N....Q..K......G**	11 % (1/9)
**VNP16**	**.....................T ...... YNK.......LQ.........D**	80 % (4/5)
	**.....................T ...... YK...N....LQ.........G**	20 % (1/5)
**RP-2**	**................A..... ....RT .GD........Q..K......D**	88 % (7/8)
	**................A..... ..G.RT .GD........Q..K......D**	12 % (1/8)
**RP-6**	**R.............P.A....T ...... .SQ........Q.....N...G**	44 % (4/9)
	**R.............P.A....T ...... .SQT.......Q.....N...G**	11 % (1/9)
	**..............P.A....T ...... .SQ........Q.....N...N**	11 % (1/9)
	**..............P.A....I ...... .SQ.......IQ.....N...G**	11 % (1/9)
	**..............P.A....T ...... .SQ.......IQ.....N...G**	11 % (1/9)
	**....V.........P.A....T ...... .SQ........Q.D...N....**	11 % (1/9)
**RP-7**	**................A....T ...... .SQ........Q.........N**	29 % (2/7)
	**................A....T ...... .NQ........Q..........**	29 % (2/7)
	**................A....T ...... .NQ........Q.........N**	14 % (1/7)
	**...........P....A....T ...... .SQ........Q.........N**	14 % (1/7)
	**................A....T ...... .NQ..N.....Q.........N**	14 % (1/7)
**RP-8**	**.....................T ...... YNL..Q.....Q.........G**	75 % (9/12)
	**.....................T ...... YNL..Q....IQ.........G**	8 % (1/12)
	**.....................N ...... YNL..Q.....Q.........G**	8 % (1/12)
	**.....................N ...... YNL..Q.....Q.........D**	8 % (1/12)
**RP-10**	**....L...........N....S ...... IEA..E.....Q..K..G..SE**	80% (4/5)
	**....L...........N....S ...... IKA..E.....Q..K..G..SE**	20% (1/5)

To assess the effect of these changes in the ability of cloned Env to induce NKp44L expression, pseudoviruses devoid of Env or carrying either BaL, NL4-3 or patient-derived Env clones were incubated with stimulated CD4 T cells isolated from healthy donors. As expected, Env-defective virions were unable to induce NKp44L expression, while BaL, NL4-3 pseudoviruses or a synthetic 3S peptide elicited a high level of NKp44L expression (Figure 3B). Selected Env clones from all VNPs induced the expression of NKp44L to a similar extent. Comparable effects were obtained using envelope clones isolated from RPs (Figure 3B) or using additional clones from individuals VNP-8 and 11 (not shown). The addition of a polyclonal anti-3S antibody significantly inhibited (p = 0.0003) this effect confirming the role of the 3S epitope (Figure 3C).

**Figure 4:**
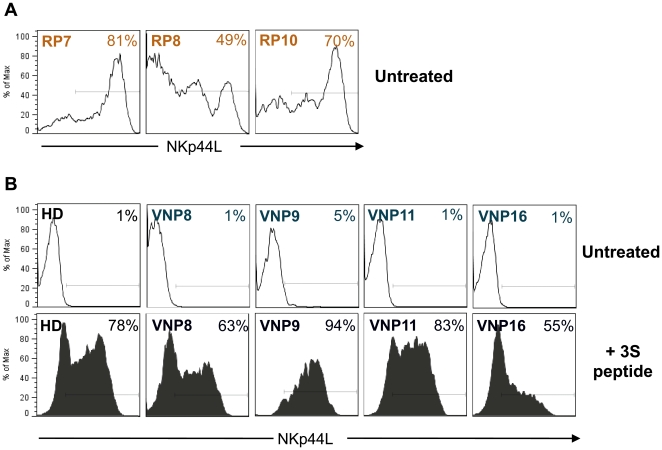
Expression of NKp44L in CD4 T cells from RPs and VNPs. The *in vivo* expression of NKp44L was assessed in gated CD3+ CD4+ cells after staining thawed PBMC from RP (panel A) and VNP patients (panel B, upper plots). A representative healthy donor was also analyzed (left plot in panel B). PBMC from VNP individuals or from the healthy donor were also incubated for 5 h in the presence of the 3S consensus peptide V (lower plots in panel B). In each plot, values indicate the % of positive cells.

We next evaluated the expression of NKp44L in available cryopreserved PBMCs from RP and VNP. CD4 T cells from three RPs who had available PBMC samples expressed high levels of NKp44L (81, 49 and 70%, respectively, Figure 4A), while very low (5%, patient 9) or undetectable cell-surface NKp44L levels were detected in CD4 T cells from VNP (Figure 4B). Remarkably, cells from VNP translocated the ligand to the cell surface after treatment with a synthetic 3S peptide (15-mer consensus peptide V) to a similar extent than cells isolated from an uninfected individual (Figure 4B). This observation along with the full capability of Env clones isolated from VNPs to induce NKp44L, suggest that humoral responses could be contributing to the protection of CD4 T cells from death in VNPs by hampering Env-induced expression of NKp44L on its surface and, hence, hindering its NK cell-mediated lysis.

### Analysis of humoral anti gp41 response

**Figure 5:**
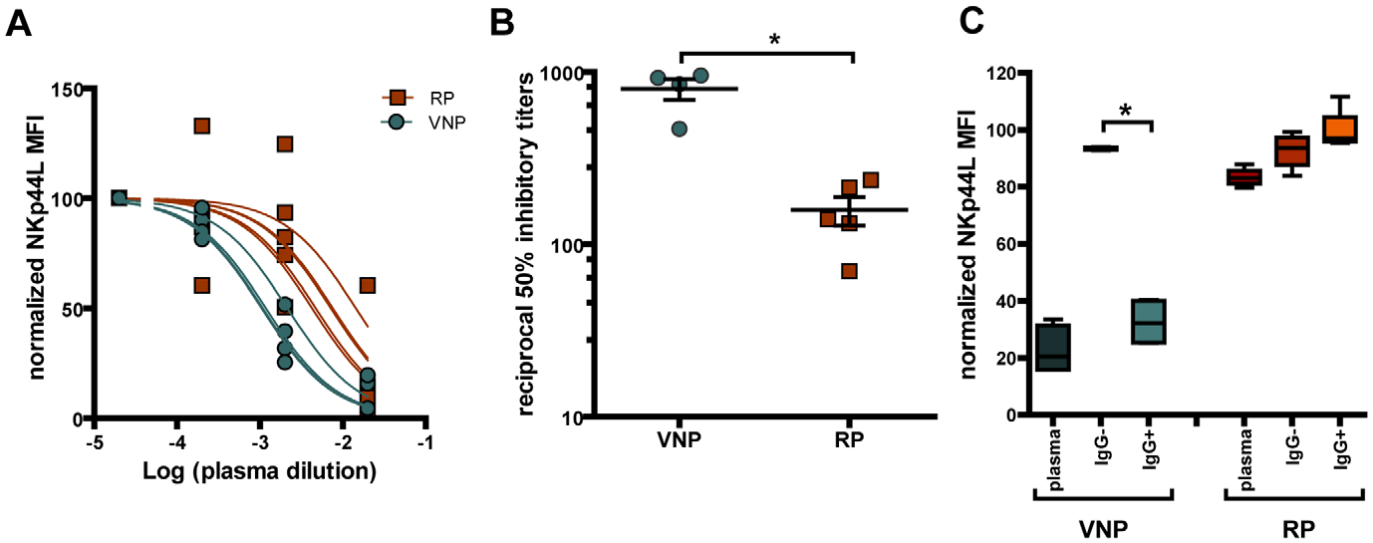
Functional analysis of anti 3S response. (A) The capacity of plasma samples from VNP patients (green symbols) and RP patients (orange symbols) to inhibit NKp44L induction by autologous pseudoviruses in CD4 T cells was tested using different dilutions of plasma (range 1/50 to 1/5000). A 100% value was given to the NKp44L expression obtained in the absence of plasma (C+) and the dose-response curves of normalized NKp44L expression are represented. (B) Reciprocal IC50 values from VNP and RP obtained after fitting the normalized individual curves from panel A are plotted. (C) Whole plasma samples (plasma, 1/100 dilution), IgG-depleted plasma (IgG-, 1/100 dilution) and purified IgG samples (IgG+, 10 µg/ml) from VNP (n = 4, left) or RP (n = 5, right) were tested for their ability to inhibit the expression of NKp44L induced by the consensus peptide V. A 100% value was given to the NKp44L expression obtained in the absence of plasma. The p value of Mann-Whitney comparisons is shown.

**Figure 6:**
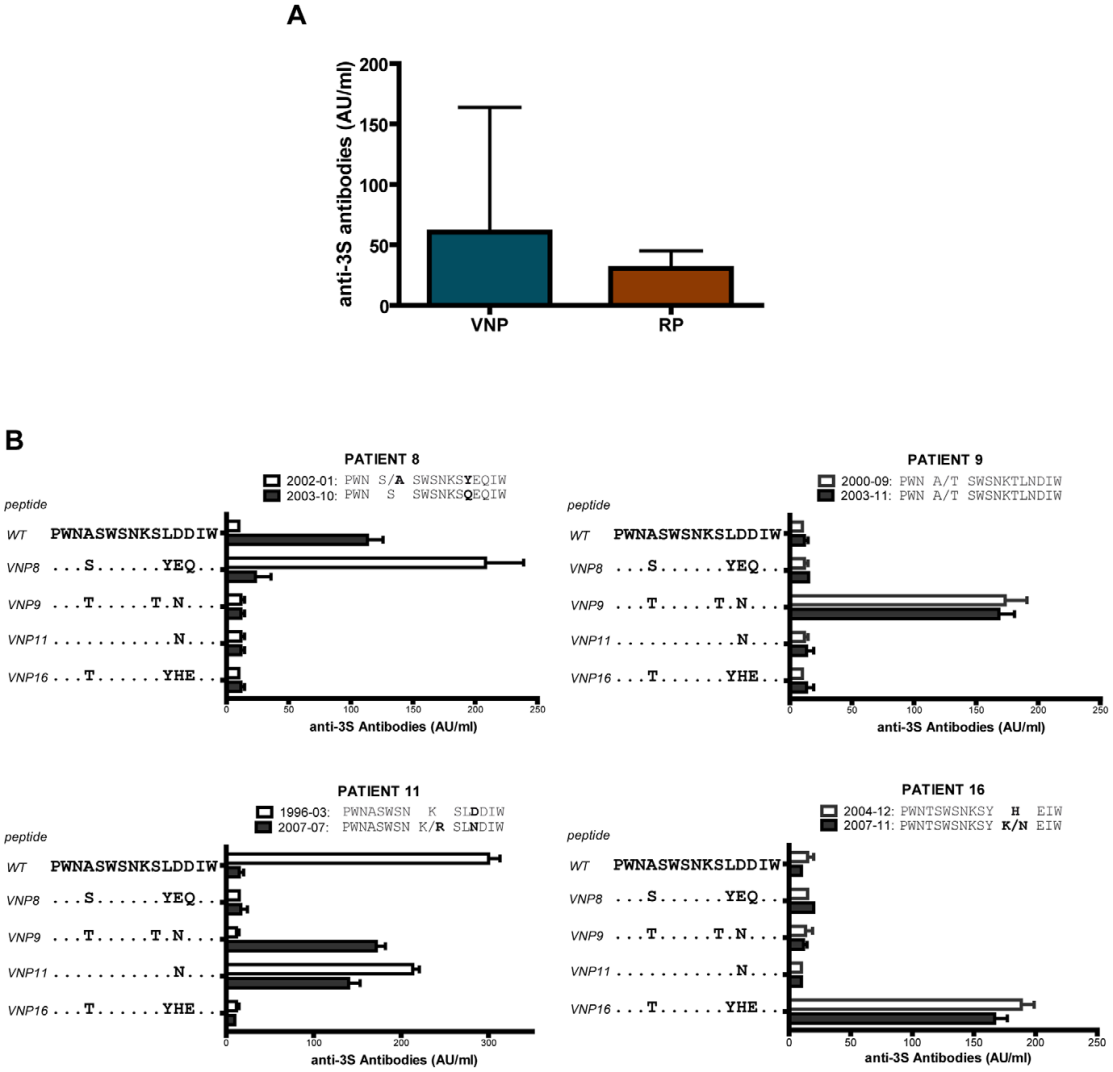
Mapping humoral responses. (A) Plasma from RP (n = 5) and VNP (n = 8) individuals were tested in triplicate for the recognition of consensus peptide V in ELISA. Data are arbitrary absorbance units per ml of plasma, boxes represent mean+/−SD. (B) Plasma from VNP patients 8 (upper left), 9 (upper right), 11 (lower left) and 16 (lower right) were assayed for the recognition of five different peptides displaying a consensus sequence covering 609–624 residues of gp41 or the equivalent patient-based envelope sequences. Two samples were analyzed for each patient, the earliest sample available (empty bars) and the sample (or closest sample for patient 9) of Env cloning (dark bars). Peptide sequences used in ELISA assays are shown in the left axis of each plot. The autologous population sequence from each patient obtained at the indicated timepoints (year-month) is also shown in top of each panel. Intrapatient sequence changes are highlighted. Data are mean+/−SD of triplicate samples.

To analyze functional responses against the 3S epitope of gp41, we evaluated the ability of serial plasma dilutions to block NKp44L induction by autologous viruses, and calculated inhibition titers (Figure 5A). All samples inhibited NKp44L induction. Importantly, when compared with RPs, plasma samples from VNPs showed significantly (p = 0.016) higher potency to block the effect of 3S epitope (Figure 5B). To confirm the role of specific anti-gp41 antibodies, we induced NKp44L expression in CD4 T cells from healthy donors with a 15-mer peptide covering the 3S sequence and evaluated the inhibitory activity of whole plasma, IgG-depleted plasma or purified IgG preparations. VNP samples showed a significant capacity to inhibit peptide-induced NKp44L expression, which was retained by IgG fraction and lost in IgG-depleted plasma. Conversely, plasma from RP individuals showed very low inhibitory activity that was not clearly associated with IgG fraction in the experimental conditions analyzed (Figure 5C). Humoral reponses were further characterized in ELISA experiments against the consensus 15-mer 3S peptide. In these experiments, first and last available plasma samples covering the non cytophativc HIV replication period of VNP were compared with plasma from RP. Consistent with functional data, RP samples showed poor peptide recognition, while VNP plasma showed higher prevalence of antibodies against this peptide (Figure 6A), although some VNP samples also showed poor reactivity in ELISA. This observation along with the variability of 3S surrounding sequences, might suggest that antibodies with a different specificity than the 3S core (SWSNKS) could also be active at blocking NKp44L induction. To test whether the variability of 3S adjacent sequences may confer some specificity to antibodies blocking the 3S motif, we designed four peptides according to the specific gp41 sequences of each VNP patient. The earliest and latest available samples from VNPs (up to 11 years timeframe) were analyzed in ELISA and their gp41 ectodomain was sequenced. Results, summarized in Figure 6B, indicate that plasma from every patient was able to recognize autologous sequences; although patient 8 switched the reactivity from the autologous to the consensus peptide overtime. Patient 11 showed parallel changes in sequence and antibody response, switching from consensus sequence (LDDIW in C terminus) to LNDIW overtime. Moreover, sera showed limited cross-reactivity; only plasma from patient 11 was able to recognize sequences from patient 9, both of them displaying an arginine at position +2 of the SWSNKS core sequence. Sequence analyses of the 3S epitope at population level (Figure 6B) confirmed mutations associated with changes in recognition patterns, and pointed to the more variable C terminal adjacent region as the key target for antibodies against the 3S flanking region in VNPs. None of the RP plasma recognized this set of peptides (data not shown). Regarding whole anti HIV neutralizing responses, VNPs had higher neutralization titers for NL4-3 or BaL isolates, but failed to mount a broad neutralizing response against HIV (Figure S1). In summary, VNPs showed an efficient sequence-dependent humoral response against the adjacent regions of the 3S epitope in the context of a rather moderate neutralizing response against HIV replication.

## Discussion

The characterization of HIV-infected patients showing discordant immunologic and virologic profiles may provide interesting clues for our understanding of HIV pathogenesis. In this study, we have characterized Env functions and Env-related immune responses in a reduced group of patients showing high viremia and either rapid CD4 T cell decay (RPs) or paradoxical high CD4 T cell counts (VNPs). Env was chosen because it is the best-defined factor altering CD4 T cell decay through coreceptor switch [9], fusogenic/hemifusogenic activity [11], [13]–[15], [33] and activation of NK-mediated lysis [20]. The analysis of these mechanisms in non-cytophatic HIV replication may help to address their individual contribution to CD4 T cell decay, although is strongly limited by the exceptionallity of these patients [17], [18].

Previous reports have described different fusion activities in envelope clones isolated from Elite controllers or pre-AIDS patients compared to patients progressing to AIDS [31], [34]. In our samples, we have also addressed the analysis of fusion capacity by isolating full-length Env clones and monitoring different functional parameters. In all cases, we were unable to detect significant differences between groups. Indeed, the percentage of functional clones, the level of Env expression (either measured by % of Env+ cells or by the Relative Fluorescence Intensity) and the Fusogenic Index were similar for VNPs and RPs. Such an irrelevant role of fusion activity in our patients compared to Lassen et al [31] may rely in a higher Env evolution in our VNPs, which show higher VL than “regular” LTNPs. In agreement with our data, similar cytopathicity in organ culture has been reported for viral clones isolated from a small group of VNPs and RPs [18]. Furthermore, the homogeneous R5 tropism of Env clones isolated from our patients ruled out any potential role of CXCR4 use in our small cohort. Although in vitro fusion assays using cell lines may differ from in vivo envelope function, our data suggest that fusion defects and viral tropism fail to explain the different outcomes of VNPs and RPs in terms of CD4 T cell loss.

Considering the deleterious role of NKp44L on CD4 T cells, which render these cells sensitive to NK lysis [20], we next analyzed the potential role of NKp44L induction by gp41. Env clones from both groups of patients showed conserved 3S epitope sequences and hence displayed full capability to induce NKp44L expression in CD4 T cells from healthy donors. However, NKp44L expression in CD4 T cells isolated from these patients showed a different profile with a lower cell-surface expression in VNP samples. Indeed, NKp44L translocation was inhibited by plasma from VNPs with significantly higher efficiency than plasma from RPs, being the purified IgG fraction responsible for this effect. Further analysis of anti-3S responses in VNP patients revealed that these individuals showed high titers of specific antibodies against sequences flanking the conserved 3S epitope. Indeed, we found that only some VNP sera tested recognized a consensus peptide due to changes in residues juxtaposed to the core sequence SWSNKS. This is the case of plasma from patient VNP-8, which strongly recognized the peptide containing a YEQ sequence in residues 620–622, which was present in the autologous virus. Consistently, patient VNP-16, whose virus shows an YKE sequence in these residues, specifically recognizes an YHE containing peptide. Interestingly, no cross reactivity between both patients was observed. A similar scenario was observed for VNP patients 9 and 11, in which the D621N change determined recognition, although in this case we observed a non-reciprocal cross-reactivity that may be related to position 612 (A or T). Moreover, the longitudinal analysis of anti-3S responses in VNPs showed that several unique specificities of antibodies inhibiting 3S triggering are present over the whole period of non-cytopathic viral replication, and hence do not seem to be the consequence of a long-term continuous antigen stimulation of B cells. It has been recently shown that the 3S epitope of gp41 mediates the NKp44L translocation to the cell surface by activating oxidative stress sensors. The 3S epitope interacts with the gC1qR on the membrane of CD4 T cells to activate a PI3K/NADPH oxidase and a p190 RhoGAP GTPase dependent pathway [21]. Thus, antibodies against the 3S epitope act by hampering 3S binding to its receptor. Taken together, these data suggest that specific anti-gp41 antibodies against variable 3S adjacent region might limit the ability of gp41 to induce NKp44L expression on CD4 cells by a similar mechanism in VNPs, although its exact mode of action remains to be defined. These antibodies may represent a compensatory defense against the gp41 pathogenicity and CD4 depletion when antibodies directed against the highly conserved 6 amino-acid 3S peptide are decreasing, as it occur normally during the disease progression [20], [22]–[24]. Consistently, very low levels of NKp44L expression were observed on the surface of CD4 T cells isolated from VNP, despite its full responsiveness to a 3S peptide and its continuous exposure to fully functional envelopes in vivo, with VL>10,000 copies/ml for more than 2 years.

By suppressing NKp44L expression, antibodies against the 3S epitope or its flanking region might contribute more to T cell protection than neutralizing antibodies. In our study, VNPs are unable to mount a broad neutralizing response against HIV and several groups have reported little, if any, impact of HIV neutralizing responses on clinical condition or progression to AIDS [35], [36]. Conversely, anti-3S antibodies, which are independent of neutralization, as this epitope is not the target of HIV neutralizing antibodies [37], may have indirect favorable effects on CD4 T cell survival [20].

In conclusion, our data, obtained in a small group of patients, support the notion that HIV Envelope glycoproteins from VNPs are functionally similar to those isolated from RPs in terms of expression, tropism, fusion and induction of NK ligands. This is consistent with other small studies [18]. However, our data point out that VNPs are able to mount strong humoral responses against a variable region flanking the 3S epitope of gp41 that block the capacity of autologous viruses to trigger surface expression of the NKp44L in CD4 T cells, a mechanism clearly related to CD4 depletion [20], [22], [24]. These antibodies may contribute to non-cytopathic HIV replication in humans along with the reported lower immune activation of these patients [17], [18] and other viral determinants outside Env [38].

## Supporting Information

Figure S1 **Neutralization activity of plasma from VNP and RP.** Neutralization capacity of plasma from VNP and RP patients was tested against pseudoviruses carrying NL43 (B), BaL (C) or primary isolates SVPB13 (C) and SVPB16 (D) envelope glycoproteins using TZM-bl reporter cell line. In figure A and B left plots represent the curves of normalized RLU for each plasma dilution in a logarithmic scale; right plots represent the reciprocal 50% inhibitory titers deduced from the normalized curves. For SVPB13 and SVPB16 no neutralization activity could be quantified (all reciprocal IC50 values were <60).
(DOC)

Table S1 **Genotypic and phenotypic characterization of the V3 loop.** ^a^ Tropism assessed in sillico by the Pssm software. Similar results were obtained using the geno2pheno software. ^b^ Tropism assessed by sensitivity to JM-2987 and TAK779 in TZM-bl cells.
(DOC)
