# Supplementary material for: Viremic HIV Infected Individuals with High CD4 T Cells and Functional Envelope Proteins Show Anti-gp41 Antibodies with Unique Specificity and Function
Source: PLoS One. 2012 Feb 1;7(2):e30330. doi: 10.1371/journal.pone.0030330 (PMC3270019; doi:10.1371/journal.pone.0030330)

**Marta Curriu et al.** *Viremic HIV infected individuals with high CD4 T cells and functional envelope proteins show anti-gp41 antibodies with unique specificity and function*.

**Figure S1. Neutralization activity of plasma from VNP and RP.** Neutralization capacity of plasma from VNP and RP patients was tested against pseudoviruses carrying NL43 (B), BaL (C) or primary isolates SVPB13 (C) and SVPB16 (D) envelope glycoproteins using TZM-bl reporter cell line. In figure A and B left plots represent the curves of normalized RLU for each plasma dilution in a logarithmic scale; right plots represent the reciprocal 50% inhibitory titers deduced from the normalized curves. For SVPB13 and SVPB16 no neutralization activity could be quantified (all reciprocal IC50 values were <60).


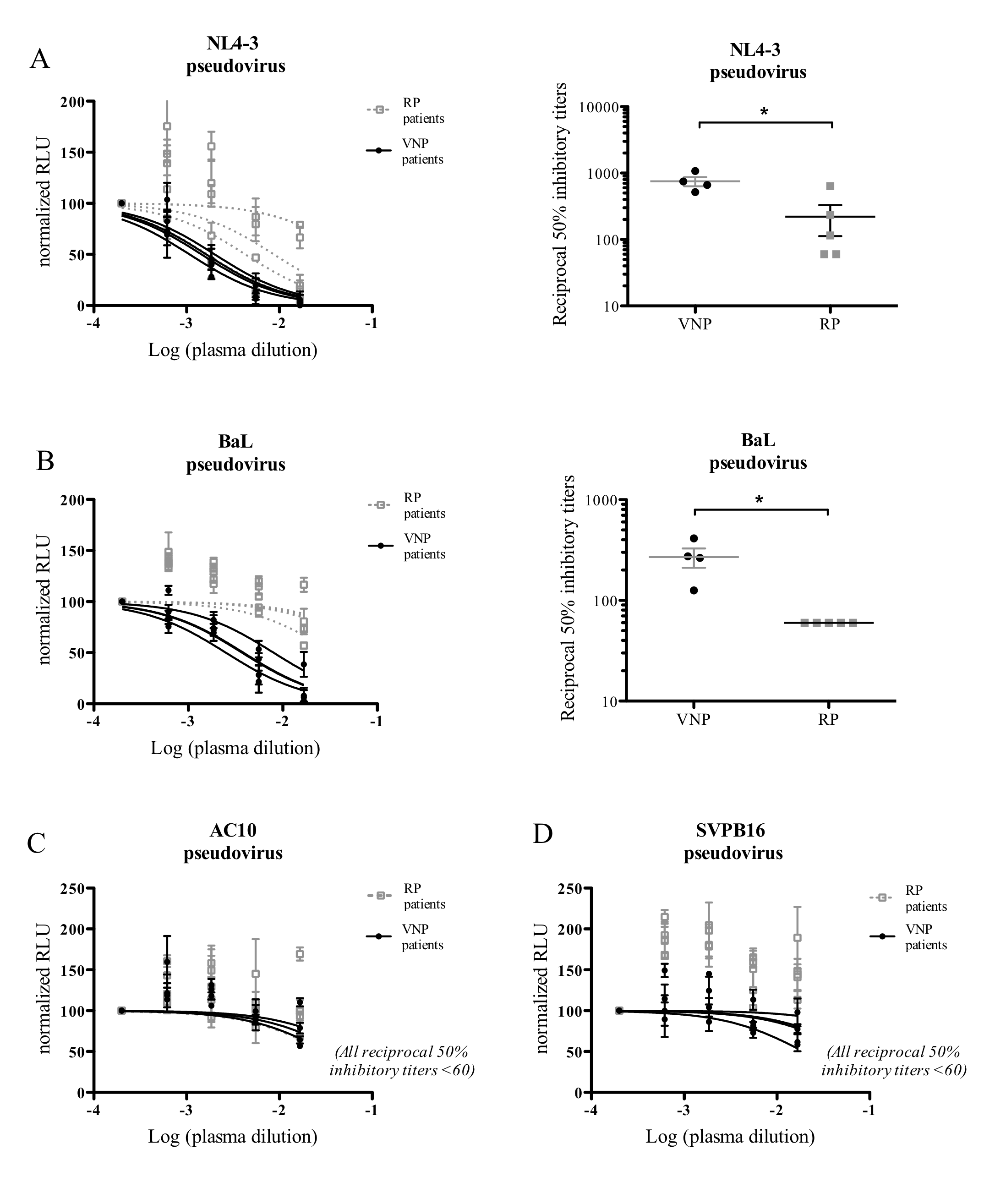

Supplement: Figure S1 — Neutralization activity of plasma from VNP and RP. Neutralization capacity of plasma from VNP and RP patients was tested against pseudoviruses carrying NL43 (B), BaL (C) or primary isolates SVPB13 (C) and SVPB16 (D) envelope glycoproteins using TZM-bl reporter cell line. In figure A and B left plots represent the curves of normalized RLU for each plasma dilution in a logarithmic scale; right plots represent the reciprocal 50% inhibitory titers deduced from the normalized curves. For SVPB13 and SVPB16 no neutralization activity could be quantified (all reciprocal IC50 values were <60). (DOC) [file pone.0030330.s001.doc]
