# Supplementary material for: Viremic HIV Infected Individuals with High CD4 T Cells and Functional Envelope Proteins Show Anti-gp41 Antibodies with Unique Specificity and Function
Source: PLoS One. 2012 Feb 1;7(2):e30330. doi: 10.1371/journal.pone.0030330 (PMC3270019; doi:10.1371/journal.pone.0030330)
Supplement: Table S1 — Genotypic and phenotypic characterization of the V3 loop. a Tropism assessed in sillico by the Pssm software. Similar results were obtained using the geno2pheno software. b Tropism assessed by sensitivity to JM-2987 and TAK779 in TZM-bl cells. (DOC) [file pone.0030330.s002.doc]

**Marta Curriu et al.** *Viremic HIV infected individuals with high CD4 T cells and functional envelope proteins show anti-gp41 antibodies with unique specificity and function*.

**Table S1. Genotypic and phenotypic characterization of the V3 loop**

|  |  |  | ***In silico*** | ***In vitro*** |
| --- | --- | --- | --- | --- |
|  |  | **Frequency** | **Pssm^a^** | **TZM-bl cells^b^** |
| ***HXB-2:*** | **CTRPNNNTRKRIRIQRGPGRAFVTIGKI-GNMRQAHC** |  |  |  |
|  |  |  |  |  |
|  |  |  |  |  |
| **VNP8** | ..........G.P.--......YAT.QVI.DI..... | 8/12 | R5 | R5 |
|  | ..........G.H.--......YAT.QVI.DI..... | 2/12 | R5 | R5 |
|  | ..........G.P.--...K..YAT.QVI.DI..... | 1/12 | R5 | R5 |
|  | ..........G.H.--......YAT.EVI.DI..... | 1/12 | R5 | R5 |
|  |  |  |  |  |
|  |  |  |  |  |
| **VNP9** | .........RSV.V--...Q.LF.T-D.I.DI..... | 7/10 | R5 | R5 |
|  | .........RSVHV--...Q.LF.T-D.I.DI.R... | 2/10 | R5 | R5 |
|  | .........RGVHV--...Q.LF.T-D.I.DI.R... | 1/10 | R5 | R5 |
|  |  |  |  |  |
|  |  |  |  |  |
| **VNP11** | ..........S.H.--......YAP.DI..DI.K... | 4/9 | R5 | R5 |
|  | ..........S.H.--......YAP.EI...I.K.Y. | 2/9 | R5 | R5 |
|  | ..........S.H.--......YAP.EV..KI..... | 2/9 | R5 | R5 |
|  | .........RS.H.--......YAP.EI...I.K... | 1/9 | R5 | R5 |
|  |  |  |  |  |
|  |  |  |  |  |
| **VNP16:** | .I..G.....S.SF--A..S..YATQG.I..I..... | 2/4 | R5 | R5 |
|  | .I..G.....S.SF--A..S..YATQ..I..I..... | 1/4 | R5 | R5 |
|  | ....G.....S.SF--A..S..YATQE.I.DI..... | 1/4 | R5 | R5 |
|  |  |  |  |  |
|  |  |  |  |  |
|  |  |  |  |  |
| **RP2** | ..........S.QV--...K.LYAT.A.I.DI..... | 6/6 | R5 | R5 |
|  |  |  |  |  |
|  |  |  |  |  |
| **RP6** | .I.......RS.HM--...ST.F.ADI.-.DI..... | 7/10 | R5 | R5 |
|  | .I.......RS.H.--...STLF.ADI.-.DI..... | 3/10 | R5 | R5 |
|  |  |  |  |  |
|  |  |  |  |  |
| **RP7** | ..........S.H.--A.....YAT.DI..DI..... | 5/7 | R5 | R5 |
|  | ..........S.H.--A..K..YAT.DI..DI..... | 2/7 | R5 | R5 |
|  |  |  |  |  |
|  |  |  |  |  |
| **RP8** | ....H.....G.H.--......YAT.D.I.DI..... | 7/12 | R5 | R5 |
|  | ..........G.H.--......YAT.D.T.DI..... | 3/12 | R5 | R5 |
|  | ..........G.H.--......YAT.D.I..I..... | 1/12 | R5 | R5 |
|  | ..........G.N.--......YAT.D.I.DI..... | 1/12 | R5 | R5 |
|  |  |  |  |  |
|  |  |  |  |  |
| **RP10** | ..........G.H.--......YAT.DI..DI.K... | 2/3 | R5 | R5 |
|  | ..........GMH.--......YAT.DI..DI.K... | 1/3 | R5 | R5 |
|  |  |  |  |  |

^a^ Tropism assessed by the Pssm software. Similar results were obtained using the geno2pheno software.

^b^ Tropism assessed by sensitivity to JM-2987 and TAK779 in TZM-bl cells
